# Automating Dental Condition Detection on Panoramic Radiographs: Challenges, Pitfalls, and Opportunities

**DOI:** 10.3390/diagnostics14202336

**Published:** 2024-10-21

**Authors:** Sorana Mureșanu, Mihaela Hedeșiu, Liviu Iacob, Radu Eftimie, Eliza Olariu, Cristian Dinu, Reinhilde Jacobs, on behalf of Team Project Group

**Affiliations:** 1 Department of Oral and Maxillofacial Surgery and Radiology, Iuliu Hațieganu University of Medicine and Pharmacy, 32 Clinicilor Street, 400006 Cluj-Napoca, Romania; 2 Department of Computer Science, Technical University of Cluj-Napoca, 400114 Cluj-Napoca, Romania; 3 Iuliu Hațieganu University of Medicine and Pharmacy, 32 Clinicilor Street, 400006 Cluj-Napoca, Romania; 4 Department of Electrical Engineering, Technical University of Cluj-Napoca, 400114 Cluj-Napoca, Romania; 5 OMFS IMPATH Research Group, Department of Imaging and Pathology, Faculty of Medicine, Katholieke Universiteit Leuven, 3000 Louvain, Belgium; 6 Department of Oral and Maxillofacial Surgery, University Hospitals Leuven, 3000 Louvain, Belgium; 7 Department of Dental Medicine, Karolinska Institute, 171 77 Stockholm, Sweden

**Keywords:** artificial intelligence, deep learning, dentistry, lesion detection, oral health, panoramic radiography

## Abstract

**Background/Objectives:** The integration of AI into dentistry holds promise for improving diagnostic workflows, particularly in the detection of dental pathologies and pre-radiotherapy screening for head and neck cancer patients. This study aimed to develop and validate an AI model for detecting various dental conditions, with a focus on identifying teeth at risk prior to radiotherapy. **Methods:** A YOLOv8 model was trained on a dataset of 1628 annotated panoramic radiographs and externally validated on 180 radiographs from multiple centers. The model was designed to detect a variety of dental conditions, including periapical lesions, impacted teeth, root fragments, prosthetic restorations, and orthodontic devices. **Results:** The model showed strong performance in detecting implants, endodontic treatments, and surgical devices, with precision and recall values exceeding 0.8 for several conditions. However, performance declined during external validation, highlighting the need for improvements in generalizability. **Conclusions:** YOLOv8 demonstrated robust detection capabilities for several dental conditions, especially in training data. However, further refinement is needed to enhance generalizability in external datasets and improve performance for conditions like periapical lesions and bone loss.

## 1. Introduction

The integration of artificial intelligence (AI) into healthcare has advanced considerably over the past decade, and the field of dentistry is no exception to this trend [1,2]. The World Health Organization estimates that nearly 3.5 billion people suffer from a form of oral disease throughout their lives [3]. This underscores a pressing need for tools to ease the burden on practitioners and improve access to oral healthcare. AI algorithms could help alleviate some of these burdens, as they have been shown to decrease workloads and increase diagnostic efficacy [4]. This may lead to earlier detection and treatment of dental pathologies and result in better oral health outcomes for patients. AI holds particular promise in dentomaxillofacial radiology, where the large volume of imaging data provides an excellent foundation for algorithm training [5].

Panoramic radiography examination is a part of the pre-treatment dental screening for patients undergoing radiotherapy for head and neck cancers [6]. Patients exposed to therapeutic radiation often experience dental complications. Radiation damage to salivary glands can result in severe xerostomia and post-radiation caries, while bone damage, potentially leading to osteoradionecrosis, may take years to develop and is frequently triggered by dental extractions [7]. Screening aims to identify and extract teeth with poor prognoses to reduce the need for post-radiation therapy extractions, which are associated with an increased risk of osteoradionecrosis [8]. Screening is also carried out before cardiac surgery, to detect and eliminate potential sources of dental sepsis before treatment onset, thereby reducing the risk of infective endocarditis [9]. The recent rise of AI presents significant potential for its integration into dental screening applications, offering opportunities to enhance inter-specialty communication, detect hard-to-see lesions, and streamline workflows.

A substantial number of AI-based decision support systems have been developed and trained on panoramic radiographs. Applications range from detection and/or segmentation of dental caries [10,11], alveolar bone loss [12,13,14,15], impacted teeth [16,17,18], maxillary sinus pathology [19,20], vertical root fractures [21], prosthetic restorations [18,22], the detection of intraosseous cysts and tumors [23,24], and osteoporosis screening [25], as well as tooth identification and numbering [26,27,28]. To the best of our knowledge, no current applications specifically target the detection of teeth at risk or needing extraction prior to radiotherapy for head and neck cancers. AI applications are widely studied in the field of dental medicine; however, despite the large amount of research, there are comparatively few clinically viable AI models. This has been attributed to limited data availability, insufficient methodological standards, and concerns about these solutions’ value, ethics, and practicality [29]. A few examples of commercially available dental charting or radiographic interpretation applications include: CranioCatch Clinic (CranioCatch, Eskişehir, Turkey), DiagnoCat (DGNCT LLC, Miami, FL, USA), VelmeniAI (Velmeni, Sunnyvale, CA, USA), and Denti.AI (Denti.AI Technology Inc., Toronto, ON, Canada). The available literature has indicated strong performance in external validation studies [30,31,32,33,34], though outcomes seem to vary significantly across different lesions or conditions.

This study aimed to train and validate an AI model for automated dental lesion detection on panoramic X-ray images. We aimed to detect a varied pool of dental pathologies and treatments; however, the focus was on the preliminary identification of teeth that posed potential risks for oncologic patients. This includes teeth presenting with periapical lesions, as well as root fragments. Our model also detects prosthetic restorations, dental implants, dental fillings, endodontic treatments, caries, periodontal bone loss, impacted teeth, root resorption, fixed orthodontic treatments, and surgical devices (i.e., osteosynthesis plates, orthodontic temporary anchorage devices).

## 2. Materials and Methods

### 2.1. Panoramic Image Selection and Dataset

The training dataset consisted of 1628 panoramic images from the Department of Maxillofacial Surgery and Radiology at the Iuliu Hațieganu University of Medicine and Pharmacy, Cluj-Napoca, Romania. The images were retrospectively collected from the department’s database of patients who visited the hospital between April 2021 and December 2023. The images were acquired using a Vatech PCH-2500 machine (Vatech, Hwaseong, Republic of Korea) and stored in jpeg format. A second set consisting of 180 radiographs was acquired from different medical centers in Cluj-Napoca, Romania, and was used as external validation. Patient consent was waived due to the retrospective nature of the study by the Ethics Committee of the Iuliu Hațieganu University of Medicine and Pharmacy, reference number 117/04.06.2024. All images were de-identified before analysis. The study was conducted in accordance with the principles of the Declaration of Helsinki.

Panoramic images of diagnostic quality were selected based on the following criteria: adult patients with permanent dentition. Images containing large cysts, tumors, metallic artifacts, blurring, or severe technique errors that would impede the radiological interpretation were excluded.

### 2.2. Image Annotation

The panoramic images were manually annotated by three calibrated researchers (S.M., M.H., and R.E.) using an open-source labeling platform (Makesense.ai v1.11.0 [35]). The inter-observer agreement was calculated using the interclass correlation coefficient (ICC), to ensure examiner reliability and achieved a value of 0.91. Bounding boxes were created for the collected images to identify the following dental conditions: prosthetic restoration, dental implant, dental filling, endodontic treatment, caries, periapical lesion, periodontal bone loss, impacted tooth, root fragment, root resorption, orthodontic treatment (brackets), and surgical devices (i.e., osteosynthesis plates, orthodontic temporary anchorage devices). Finally, the annotations were exported in the YOLO label format.

### 2.3. Preprocessing, Architecture, Training, and Evaluation

The internal dataset was split into 1375 training images, 153 validation images, and 100 testing images. An additional 180 radiographs comprised the external validation set.

The architecture used in this study was YOLO-v8 [36], a variant of the YOLO (You Only Look Once) object detector, released in January 2023. All images were resized to 1024 × 1024 pixels before being input into the model. The final model was trained over 75 epochs using the entire training dataset. A batch size of 8 was chosen, meaning the model updated its weights after every 8 images. A patience parameter of 5 epochs was set, ensuring that if the model did not show improvement within 5 consecutive epochs, the training would be halted and the best weights would be restored.

The accuracy of the model was assessed using the following metrics: F1 score, precision, and recall. The formulas for these metrics are provided in Table 1.

## 3. Results

During training, the best model achieved the following results: F1 score = 0.6 with 0.275 confidence, and recall = 0.657 for mAP@50. Although a 0.275 confidence could be considered low for other CNN architectures, in YOLO architectures, 0.3 is the recommended threshold. For the external validation set, we obtained the following results: F1 score = 0.47 with 0.192 confidence, and recall = 0.451 for mAP@50. Table 2 shows the performance results, in terms of precision and recall, for each class on the internal validation set, compared to the ground truth. Table 3 shows the performance on the external validation set. The F1-confidence curve and the precision-recall curve are provided in Appendix A.

During the training epochs, the metrics used were box_loss, class_loss, and dfl_loss (distribution focal loss) for both training and validation data.

**Figure 1:**
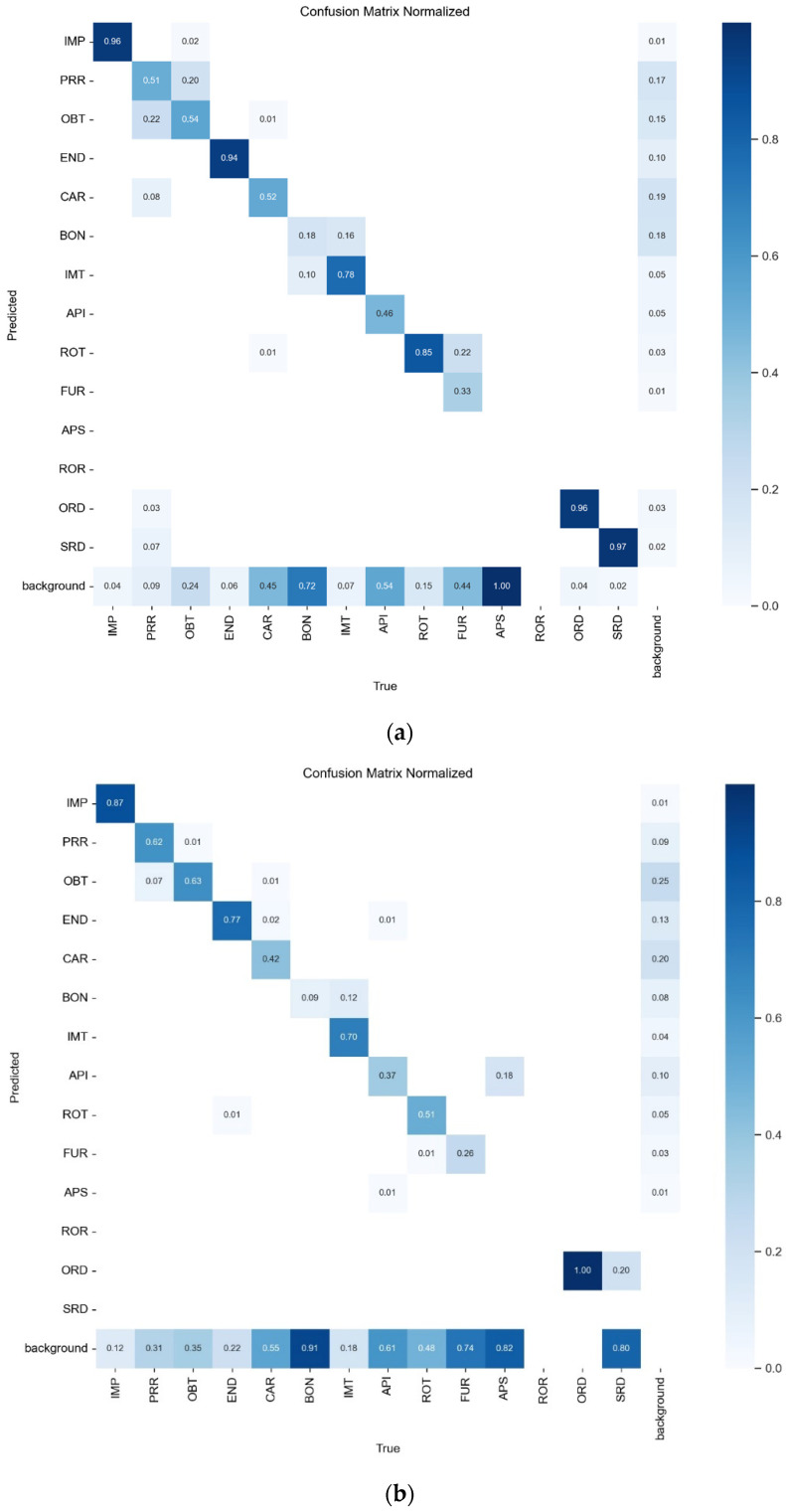
Normalized confusion matrix for the internal (**a**) and external (**b**) datasets.

**Figure 2:**
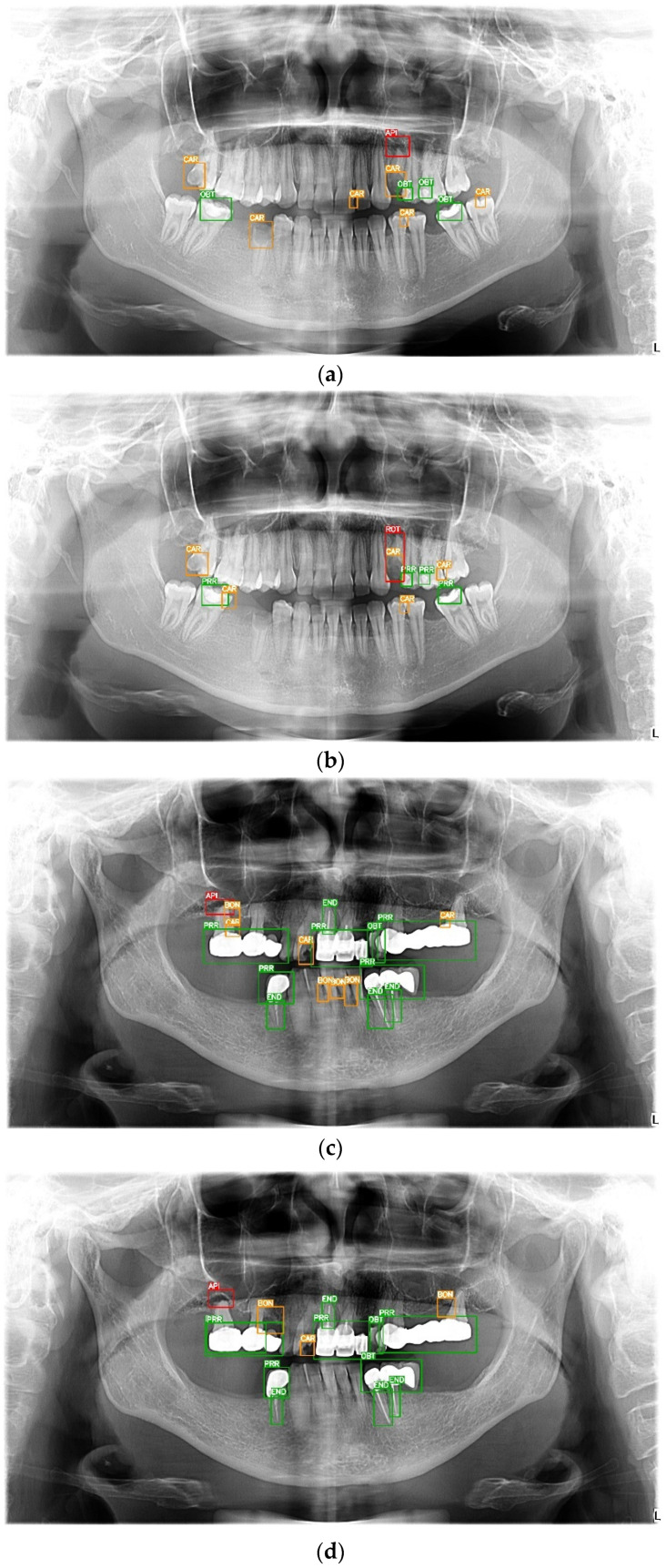
Comparison between ground truths (**a**,**c**) and predicted classes (**b**,**d**). Green labels represent dental treatments, yellow – moderate-risk lesions, and red – high-risk lesions, the target of pre-radiotherapy screening. IMP—implant; PRR—prosthetic restoration; OBT—dental filling; END—root canal treatment; CAR—carious lesion; BON—alveolar bone loss; IMT—impacted tooth; API—periapical lesion; ROT—root fragment; FUR—furcation lesion; ORD—orthodontic treatment; SRD—surgical device; APS—apical surgery.

The normalized confusion matrix for the internal and external datasets is presented in Figure 1. Figure 2 illustrates a side-by-side comparison of the model’s predictions and corresponding ground truths.

## 4. Discussion

In this study, we evaluate the results of YOLO-v8 for the detection of various dental pathological situations and treatments. During training, the algorithm showed decent performance in terms of precision, recall, and F1 score for the detection of implants, endodontic treatments, orthodontic devices, and surgical devices. Among the oral pathologies examined, impacted teeth, root fragments, and periapical lesions were the most reliably detected. The model’s evaluation metrics showed a decline when applied to the external dataset, showing a need for improvement in terms of generalizability.

Bonfanti-Gris et al. [37] evaluated the diagnostic performance of three versions of YOLO (YOLOv5, YOLOv7, and YOLOv8), in terms of object detection and segmentation, on a multiclass panoramic dataset. They found that the latest versions of YOLO showed better results for both tasks. YOLOv8 supports the following computer vision tasks: object detection, segmentation, pose estimation, tracking, and classification [36]. Our team employed YOLOv8 for object detection, as it demonstrates improved throughput compared to earlier versions while maintaining a similar number of parameters [38]. Additionally, it shows enhanced object detection performance, particularly for smaller objects, owing to the loss functions (CIoU and DFL loss functions for bounding box loss and binary cross-entropy for classification loss) [39].

### 4.1. Detection of Dental Treatments

The automated detection of dental restorations and treatments has been proposed as a first step toward the later AI-based diagnosis of dental pathology [40]. Similar to our findings, the study by Bonfanti-Gris et al. [37] faced difficulties in identifying and differentiating between coronal restorations and crowns/pontics; however, we did not face this problem with endodontic treatments. Similar trends have been observed in other studies. One study used YOLOv4 to detect prosthetic restorations on a large panoramic dataset [22]. They found that the model struggled to detect crowns, partly due to the inclusion of images with artifacts and superpositions. Similarly, our study did not exclude such images. The use of data from multiple centers may be a key factor contributing to our performance being significantly lower than that reported in the literature. Abdalla-Aslan et al. [40] developed a machine learning algorithm for automatically detecting and classifying several dental restorations. Results for amalgam fillings, crowns, implants, and cores were high. However, composite fillings and root canal treatments had lower detection rates due to their similarity to normal teeth and narrow structures. In the case of our study, implants and endodontic treatments were reliably detected.

A novel aspect of our paper is the inclusion of labels for surgical devices (in this case osteosynthesis plates) and orthodontic devices (including brackets, palatal expanders, and TADs). The model demonstrated robustness in accurately detecting these elements.

### 4.2. Detection of Dental Pathology

The detection of impacted teeth has been extensively studied in the literature. One study achieved high precision and recall using YOLOv3 for the detection of impacted third molars [16]. Another study by Kuwada [20] showed that DetectNet and AlexNet had the potential for detecting impacted supernumerary teeth in the maxillary incisor region. Another framework proposed by Zhu et al. [41] also diagnosed impacted teeth with high sensitivity and specificity. Our dataset included a broader range of dental impaction cases, encompassing not only impacted molars but also canines and premolars. While the detection of impacted teeth yielded the highest results among the various dental pathological conditions, our model performance was still lower than reported in other studies.

Periapical lesions are common dental pathologies normally resulting from apical periodontitis [42]. While our model performed surprisingly well on the training dataset, performance dropped on the external images. A clinical validation of the DiagnoCat software showed high specificity and reproducibility for lesion detection in panoramic radiographs, but limited reliability for the assessment of caries and periapical lesions [30]. Another study on DiagnoCat performed this detection on periapical radiographs [34], achieving a 0.92 F1 score. Kazimierczak et al. [43] found that periapical lesion detection had higher sensitivity and specificity on CBCT compared to panoramic radiography. The difficulties in AI-based detection of periapical lesions are reflected by the limitations of available diagnostic methods, with panoramic radiography, although common, having the lowest sensitivities [44]. Periapical radiographs result in higher accuracies, but limitations such as superimpositions and distortions [45] still affect the diagnostic process. CBCT overcomes these limitations but is associated with increased false-positive results [46]. Furthermore, the increased radiation doses additionally prevent CBCT from being used routinely. Given these considerations, we see significant value in AI-based panoramic detection for periapical lesions. It offers a reliable second opinion and has the potential to identify early or small lesions that may otherwise be overlooked.

Several tools have been developed to assess periodontal bone levels. One study [47] trained a deep learning model to evaluate bone loss on panoramic radiographs, achieving dentist-level accuracy. Kim et al. [48] improved upon it with a larger dataset, transfer learning, and clinical knowledge in their DeNTNet model. Chang et al. [49] introduced a method to detect and classify bone loss into periodontitis stages, though it relied solely on radiographic data. In our study, bounding box annotation was not appropriate for these lesions. Instead, other labeling strategies may be necessary to achieve more robust results.

Carious lesion detection had some of the lowest precision and recall scores. Other studies have faced similar challenges in detecting caries on panoramic radiographs [30,41,50], most likely due to variations in the position, shape, and extension of these lesions. Another reason might be related to observer under-detection of incipient and/or interproximal caries on radiographic findings [51]. This also explains the large number of false negatives.

### 4.3. Panoramic Radiographs

Panoramic radiography is one of the most common diagnostic tools in dentistry. It provides a two-dimensional overview of the teeth and jaws. While being a valuable adjunct for diagnosis, it presents with certain pitfalls: variable image contrast (influenced by the selection of the appropriate peak kilovoltage), image magnification, superimpositions and distortions, the image’s tomographic character (reduced visibility of structures located outside the focal through), and susceptibility to technical errors [52,53]. Therefore, diligence and a systematic approach to their interpretation are required [54]. We addressed this through meticulous calibration and training of the examiners; however, it is important to recognize that panoramic radiography may not be the most optimal diagnostic examination for some conditions. Furthermore, the variability of panoramic radiograph quality may also constitute a technical issue for AI models trained on these images.

### 4.4. Potential for Pre-Radiation Therapy Dental Screening

Head and neck cancer patients often experience a variety of treatment-related oral symptoms, including xerostomia, thickened secretions, mucosal sensitivity, dental caries, periodontal disease, infections, odynophagia, and osteoradionecrosis [55]. Pre-radiation dental screening and interventions are necessary to manage the risk of osteonecrosis [8]. The primary goal is to prevent post-radiation dental extractions when the risk of osteoradionecrosis is significantly higher [56]. Some AI models in the literature detect multiple dental conditions, including compromised teeth [32,50]. However, no studies focus specifically on this area. Regarding our model’s potential for dental screening, we achieved strong results in detecting periapical lesions and root fragments. One study emphasizes the necessary to evaluate even more pathologies, including dental caries, pulpal and periapical disease, root resorption, periodontal disease, tooth mobility, and tooth impactions [57]. Although defining tooth prognosis is a difficult task [58], we believe that AI-based screening could play a valuable role in this process in the future.

### 4.5. Practical Concerns for Clinical Implementation

Although AI has the potential to improve many aspects of dental practice, its translation into practice has been slow [59]. Reasons for this include concerns over data privacy, the lack of methodological transparency of AI methods (“black-box”), health inequity issues due to potential data biases, and underdeveloped government regulations [60]. In light of these challenges, van der Vegt et al. introduced SALIENT [61], a provisional implementation framework, which aims to aid healthcare providers in navigating the steps required to integrate AI into clinical practice. Along with new standards and regulations, physicians require training in machine learning to build the skills necessary to interpret the outputs of algorithmic decision support systems.

### 4.6. Limitations and Future Research

This study faced limitations due to the small dataset and the imbalanced classes. The latter was anticipated given the study’s focus and reflects the varying frequencies of different dental pathologies [62]. Overfitting was a concern in the starting phase. Still, to our advantage, the model exhibited overfitting only in a few classes (i.e.: carious lesion vs. obturation, preferring obturation over the other). On the other hand, the model presented an underfitting behavior for the bone resorption class due to the limited context provided by the annotation bounding boxes. This issue will be addressed in the future by considering a broader context while training and potentially employing a different annotation strategy. Another class for which the model underfitted was apical surgery, because of the limited data regarding that lesion. We aim to address this in future studies by expanding the training dataset and incorporating images from various centers to enhance its generalizability.

## 5. Conclusions

The present method achieved reliable results in detecting several oral conditions, including impacted teeth, root fragments, periapical lesions implants, endodontic treatments, orthodontic devices, and surgical devices. Our results also highlight AI’s potential to aid in pre-radiation therapy dental screening. Despite the promising findings, the inherent limitations of this study and the technical challenges associated with using panoramic radiographs for AI-based detection require that the results be interpreted with caution. Further research will need to improve generalizability, increase the sample size for validation, tackle annotation issues, and improve the model’s capability to detect critical conditions. Our results highlight the need for external, as well as clinical validation in AI studies, before their integration into dental practice.

## Figures and Tables

**Table 1:** Evaluation metrics.

Metric	Formula	Definition
Precision	$\frac{TP}{TP+FP}$	The fraction of relevant instances among all retrieved instances.
Recall	$\frac{TP}{TP+FN}$	The fraction of retrieved instances among all relevant instances.
F1	$\frac{TP}{TP+\frac{1}{2}(FP+FN)}$	F1 score combines precision and recall into a single metric.

TP—true positive rate; FP—false positive rate; FN—false negative rate.

**Table 2:** Model performance on the internal dataset.

Class	Images	Instances	Precision	Recall
all	100	1369	0.625	0.587
IMP	100	25	0.878	0.96
PRR	100	89	0.311	0.483
OBT	100	391	0.778	0.506
END	100	267	0.879	0.899
CAR	100	141	0.626	0.511
BON	100	118	0.285	0.169
IMT	100	58	0.722	0.759
API	100	80	0.779	0.412
ROT	100	33	0.649	0.818
FUR	100	18	0.584	0.235
APS	100	4	0	0
ORD	100	25	0.718	0.917
SRD	100	120	0.92	0.964

IMP—implant; PRR—prosthetic restoration; OBT—dental filling; END—root canal treatment; CAR—carious lesion; BON—alveolar bone loss; IMT—impacted tooth; API—periapical lesion; ROT—root fragment; FUR—furcation lesion; ORD—orthodontic treatment; SRD—surgical device; APS—apical surgery.

**Table 3:** Model performance on the external validation set.

Class	Images	Instances	Precision	Recall
all	180	2980	0.539	0.498
IMP	180	16	0.619	0.875
PRR	180	206	0.659	0.639
OBT	180	897	0.677	0.666
END	180	528	0.734	0.777
CAR	180	296	0.466	0.439
BON	180	680	0.437	0.107
IMT	180	111	0.765	0.793
API	180	106	0.367	0.396
ROT	180	93	0.561	0.57
FUR	180	19	0.155	0.211
APS	180	17	0	0
ORD	180	6	0.563	1
SRD	180	5	1	0

IMP—implant; PRR—prosthetic restoration; OBT—dental filling; END—root canal treatment; CAR—carious lesion; BON—alveolar bone loss; IMT—impacted tooth; API—periapical lesion; ROT—root fragment; FUR—furcation lesion; ORD—orthodontic treatment; SRD—surgical device; APS—apical surgery.

**Figure A1:**
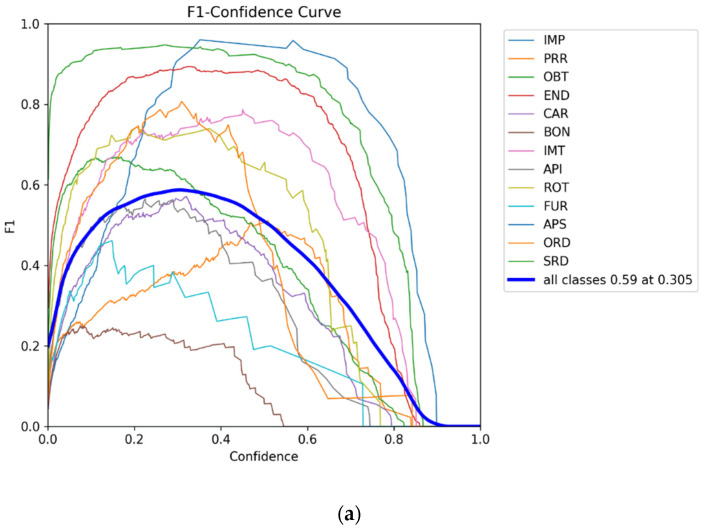

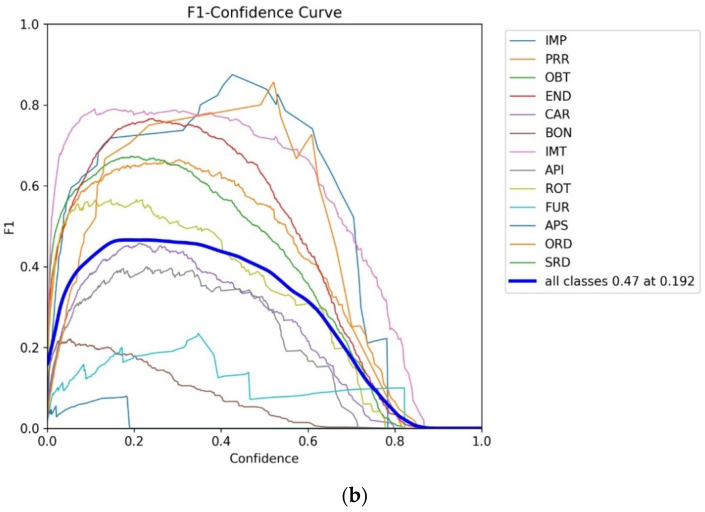
F1–confidence curves on the internal (**a**) and external (**b**) datasets.

**Figure A2:**
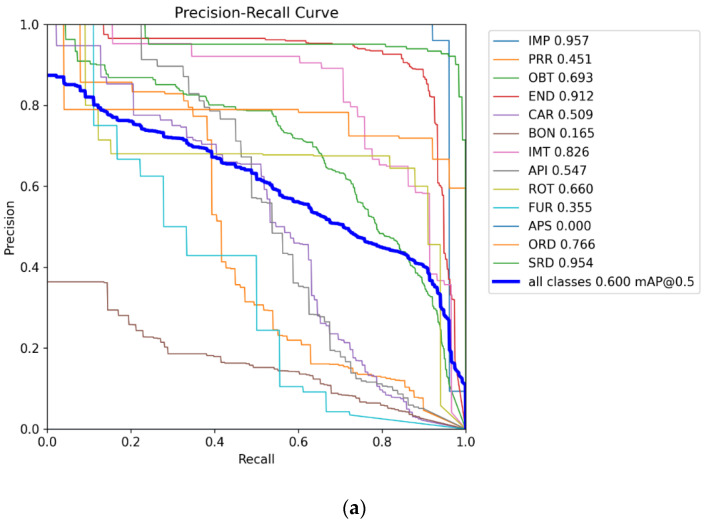

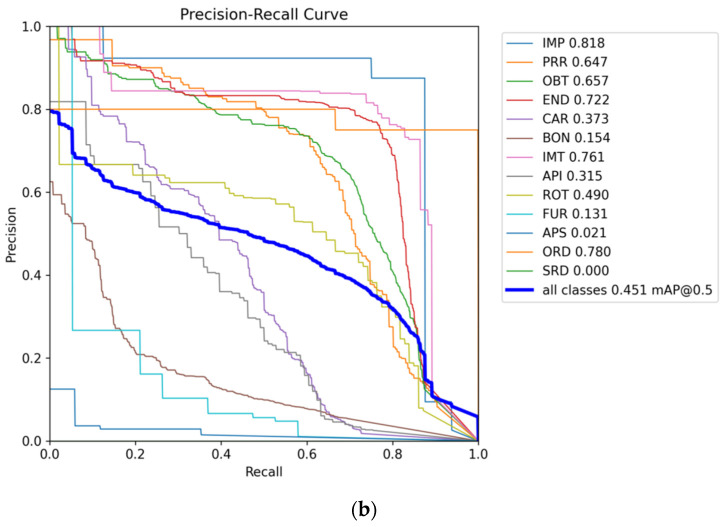
Precision–recall curves on the internal (**a**) and external (**b**) datasets.

## Data Availability

Data are available upon reasonable from the corresponding author.
